# Sex Differences in Recombination in Sticklebacks

**DOI:** 10.1534/g3.118.200166

**Published:** 2018-04-09

**Authors:** Jason M. Sardell, Changde Cheng, Andrius J. Dagilis, Asano Ishikawa, Jun Kitano, Catherine L. Peichel, Mark Kirkpatrick

**Affiliations:** * Department of Integrative Biology, University of Texas at Austin, Texas 78712; † Department of Population Genetics, National Institute of Genetics, Mishima, Shizuoka 411-8540, Japan; ‡ Institute of Ecology and Evolution, University of Bern, 3012, Switzerland

**Keywords:** recombination, heterochiasmy, genomic differentiation, chromosome center biased differentiation, sex chromosomes

## Abstract

Recombination often differs markedly between males and females. Here we present the first analysis of sex-specific recombination in *Gasterosteus* sticklebacks. Using whole-genome sequencing of 15 crosses between *G. aculeatus* and *G. nipponicus*, we localized 698 crossovers with a median resolution of 2.3 kb. We also used a bioinformatic approach to infer historical sex-averaged recombination patterns for both species. Recombination is greater in females than males on all chromosomes, and overall map length is 1.64 times longer in females. The locations of crossovers differ strikingly between sexes. Crossovers cluster toward chromosome ends in males, but are distributed more evenly across chromosomes in females. Suppression of recombination near the centromeres in males causes crossovers to cluster at the ends of long arms in acrocentric chromosomes, and greatly reduces crossing over on short arms. The effect of centromeres on recombination is much weaker in females. Genomic differentiation between *G. aculeatus* and *G. nipponicus* is strongly correlated with recombination rate, and patterns of differentiation along chromosomes are strongly influenced by male-specific telomere and centromere effects. We found no evidence for fine-scale correlations between recombination and local gene content in either sex. We discuss hypotheses for the origin of sexual dimorphism in recombination and its consequences for sexually antagonistic selection and sex chromosome evolution.

Recombination has critical consequences for evolution. Considerable research has focused on how sex-averaged recombination rates affect adaptation, population differentiation, and genome evolution (Hill and Robertson 1966; Felsenstein 1974; Charlesworth and Charlesworth 2000; Otto 2009; Keinan and Reich 2010; Webster and Hurst 2012; Burri *et al.* 2015; Ritz *et al.* 2017). However, males and females often differ strikingly in overall rate of recombination (heterochiasmy), as well as in the broad- and fine-scale distributions of crossovers along their chromosomes (Burt *et al.* 1991; Broman *et al.* 1998; Sakamoto *et al.* 2000; Lenormand 2003; Kong *et al.* 2010; Brandvain and Coop 2012). For example, in many species, males exhibit highly elevated recombination rates at the ends of chromosomes, while females exhibit more uniform local recombination rates (Singer *et al.* 2002; Rexroad *et al.* 2008; Kong *et al.* 2010; Paigen and Petkov 2010; Wong *et al.* 2010; Giraut *et al.* 2011; Jones *et al.* 2013; Brelsford *et al.* 2016; Smeds *et al.* 2016). A full understanding of the evolution of recombination therefore must account for these sex-specific patterns.

Theory shows that differences in recombination between sexes can have important consequences for several evolutionary processes. Sex-specific recombination rates affect the resolution of sexually antagonistic selection (Connallon and Clark 2010). Because recombination between homologous chromosomes is restricted to one sex, sex-specific recombination rates are relevant when sexually antagonistic selection drives sex chromosome turnover and the shutdown of recombination between sex chromosomes (Charlesworth and Charlesworth 1980; van Doorn and Kirkpatrick 2007; van Doorn and Kirkpatrick 2010). Sex-specific recombination rates and patterns will also influence patterns of introgression when the fitness of hybrids differs between sexes, as commonly occurs (Haldane 1922).

We conducted the first genome-wide analysis of sex-specific differences in recombination in threespine sticklebacks (genus *Gasterosteus*), an emerging model system for studies of population divergence, speciation, host-parasite interactions, and sex chromosome evolution (Mckinnon and Rundle 2002; Kitano *et al.* 2009; Barber and Scharsack 2010; Jones *et al.* 2012; Peichel and Marques 2017). Glazer *et al.* (2015) and Roesti *et al.* (2013) used reduced-representation sequencing to produce sex-averaged linkage maps for threespine sticklebacks, which revealed elevated recombination toward the ends of chromosomes. These studies, however, did not characterize sex-specific differences in recombination (Berner 2013). Cuñado *et al.* (2002) found that synaptonemal complexes were 5% longer in male threespine sticklebacks, suggesting that recombination rates may be greater in males than females, but lengths of synaptonemal complexes are not always correlated with recombination map lengths in teleost fish (Campos-Ramos *et al.* 2009). Rastas *et al.* (2015) found that map lengths are greater in females than males in ninespine sticklebacks (*Pungitius pungitius*), which diverged from threespine sticklebacks at least 15 million years ago (Bell *et al.* 2009; Aldenhoven *et al.* 2010).

Sex differences in recombination might have several impacts on stickleback evolution. First, the family of stickleback fishes (Gasterosteidae) is notable for high rates of turnover in their sex chromosomes (Ross *et al.* 2009). If recombination differs substantially between sexes, any change in sex linkage will greatly alter the recombination maps of the old and the new sex chromosomes. Second, genetic differentiation between ecotypes of threespine stickleback is correlated with sex-averaged recombination rate (Roesti *et al.* 2012; Berner and Roesti 2017; Samuk *et al.* 2017). This results in an enrichment of highly differentiated regions in the center of chromosomes, a pattern termed “chromosome center biased differentiation” by Roesti *et al.* (2012). There are two unresolved questions about this finding. One is whether this pattern is predominantly driven by recombination patterns in males, as expected based on observations from other taxa. Second, although Roesti *et al.* (2012) and Berner and Roesti (2017) suggest that chromosome center biased differentiation is not a byproduct of reduced recombination around the centromere, that hypothesis has not been tested quantitatively.

We directly identified crossovers (COs) by sequencing whole genomes of 15 families, each consisting of a Japan Sea stickleback (*G. nipponicus*) father, a threespine stickleback (*G. aculeatus*) mother, a son, and a daughter. These data yielded fully-phased haplotypes of the gametes that were transmitted to the offspring. Comparison of gametes inherited by siblings allowed us to identify crossover events. The hybrid cross design was used because the increased genetic variation between the parents increases the resolution. A disadvantage of our design (which was crafted for an unrelated study) is that sex differences are conflated with interspecific differences in recombination. We therefore also analyzed historical sex-averaged recombination rates using a bioinformatic approach, which supported the conclusion that sex-averaged recombination patterns are largely conserved across stickleback species, as has been found in previous studies of more distantly-related species (Roesti *et al.* 2013; Glazer *et al.* 2015; Rastas *et al.* 2015). We return to this issue in the Discussion.

We found that overall map lengths are greater in females than males, primarily due to more frequent double COs in females. Most COs in males fall at the end of long chromosomes arms, which results from an enrichment of COs near the telomeres and suppression of recombination near the centromeres. In contrast, COs in females are much more evenly distributed along chromosomes, with a much smaller effect of telomeres and centromeres on recombination. Genomic differentiation between threespine sticklebacks and sympatric Japan Sea sticklebacks is highly correlated with sex-averaged recombination rate, and is dependent on distances from both centromeres and telomeres. Finally, COs are correlated with GC content in both sexes, but more so in males than in females, likely because GC content is enriched near chromosome ends where male recombination tends to occur.

## Materials and Methods

### Sampling and Sequencing

We sampled Japan Sea sticklebacks and marine threespine sticklebacks from Akkeshi and Biwase in eastern Hokkaido, Japan (Kitano *et al.* 2007; Ravinet *et al.* 2014) in 2012, 2013, and 2016. We made 15 hybrid crosses between Japan Sea males and threespine females, and raised the crosses at the Fred Hutchinson Cancer Research Center in Seattle, USA (Institutional Animal Care and Use Protocol 1575) and the National Institute of Genetics in Mishima, Japan (Institutional Animal Care and Use Protocol 28-11). For each of the 15 crosses, genomic DNA was extracted from fin clips of the father, the mother, one son, and one daughter using the Qiagen DNeasy Blood and Tissue kit.

Paired-end whole genome sequencing was conducted on eight families (32 individuals) using the Illumina HiSeq 2500 platform at the Fred Hutchinson Cancer Research Center (resulting in 100 bp reads) and seven families (28 individuals) using the Illumina HiSequation 3000 platform at the University of Bern (resulting in 151 bp reads). Mean sequencing coverage was 28X per individual. One female was used as the mother in three of the crosses. To avoid pseudoreplication, we included two randomly-selected gametes from this female and excluded the other four. Another mother was identified as an F_1_ hybrid following genomic sequencing, and we removed her from our study because we were unable to identify any crossovers in her gametes. This left us with phased haplotypes from 30 male gametes and 24 female gametes.

### Sequence Assembly, SNP Calling, and Identification of Crossovers

Poor quality reads and overrepresented sequences were identified and removed using FastQC v11.5 (Andrews 2010). Raw paired-end reads then were mapped to the most recent threespine stickleback reference genome (Glazer *et al.* 2015) using bwa v7.12 (Li and Durbin 2010) and sorted based on the reference assembly using SAMtools v1.3 (Li *et al.* 2009). SNPs were identified using mpileup in SAMtools v1.3 (Li *et al.* 2009). We also performed mapping using Bowtie2 v2.3.2 (Langmead and Salzberg 2012) and variant calling using GATK v3.5 (Van der Auwera *et al.* 2013) to confirm that results were quantitatively similar under different methods. All indels and SNPs with more than two alleles in the population were removed using VCFtools v1.15 (Danecek *et al.* 2011).

**Figure 1:**
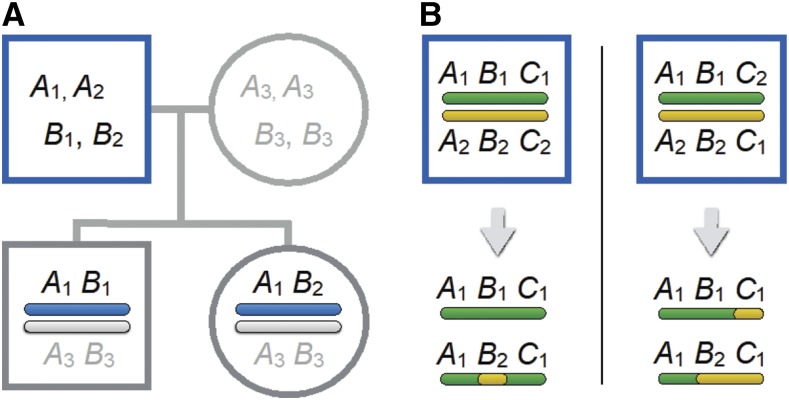
(A) The experimental design. Sequencing families with four individuals allows us to reconstruct the phased gametes that parents contributed to each offspring. Recombination events are inferred when different pairs of alleles are linked in the gametes passed to the two offspring by the same parent. The father is heterozygous at loci *A* and *B*, but we do not know the phase of his alleles. He contributed *A*_1_*B*_1_ to his son and *A*_1_*B*_2_ to his daughter, implying that a crossover occurred between SNPs at sites *A* and *B* in one of the two gametes he passed to those offspring. (B) The pairs of gametes shown at the bottom of each panel imply two recombination events. Depending upon the phase of the alleles in the parent, these gametes could either have resulted from a double crossover in one gamete and no recombination in the other (left panel) or from a single crossover in each of the two gametes (right panel).

The two gametes for each offspring were phased using the PhaseByTransmission algorithm in GATK v3.5 (Van der Auwera *et al.* 2013). This approach compares parental SNP genotypes to offspring SNP genotypes to identify the haplotype of the sperm and egg that produced the offspring (Figure 1A). A site is informative if the offspring is heterozygous at that SNP and at least one parent is not. Phased gametic haplotypes were filtered for quality by removing SNPs in which the genotype quality (GQ) score was less than 999, or where the phasing accuracy (TP) was less than 60. Use of different filtering criteria did not affect the general patterns of CO distribution that we identified. SNPs were then categorized based on whether the two offspring inherited the same allele or different alleles from a given parent.

A crossover manifests as a change from a segment of chromosome in which the two offspring inherited the same alleles to one where they inherited different alleles (Figure 1A). This pattern allows us to determine where a crossover occurred, but we cannot distinguish which gamete is recombinant. We also cannot distinguish between the case in which one crossover occurred in each of the gametes inherited by siblings from the same parent and the case in which a double crossover occurred in one of the gametes but the other gamete did not crossover (Figure 1B).

We identified transitions in allele sharing patterns between siblings using the Changepoint v2.2.2 R package (Killick and Eckley 2014). Each CO is localized to a region between two SNPs, and we refer to this region as the “CO interval”. We confirmed that these results matched plots of the allele sharing patterns (in which siblings’ gametes were given a binary code denoting whether they had the same or different alleles for each SNP and averaged over 100kb windows). Any putative COs identified by the Changepoint algorithm that did not result in an unambiguous change in this allele sharing parameter from less than 0.1 to greater than 0.9 or vice versa were excluded as potential bioinformatics noise (likely caused by phasing errors). We excluded pairs of putative COs separated by less than 400 kb because these were likely to represent gene-conversion events or mapping errors (Smeds *et al.* 2016). Similarly, we also excluded putative COs that were within 400 kb of the chromosome end, due to uncertainty in whether they represent COs or gene conversion events, as well as concerns about the algorithm’s accuracy in detecting change points at the beginnings and ends of series. Thus, we conservatively include only those COs supported by the strongest evidence.

To test whether patterns of recombination rates differed substantially between the two species, we used a population genetics approach implemented in LDhelmet v1.7 (Chan *et al.* 2012) to estimate historic sex-averaged recombination. The data comprised one phased gamete from each cross, and the phased alternate haplotype from the parental genome. SNPs were removed if any individual was not genotyped or if mean read depth was less than 15X or greater than 67X. These coverage thresholds were chosen based on the distribution of coverage across SNPs. We assumed a population-scaled mutation rate of θ = 10^−3^, based on estimates of effective population size (Caldera and Bolnick 2008; Ravinet *et al.* 2017) and mutation rate (Guo *et al.* 2013) for sticklebacks. We used default values for all other parameters. We report only the relative patterns of recombination along chromosomes rather than the estimated recombination rates. The latter estimates are highly sensitive to the effective population size used in the analyses, limiting the accuracy of this approach for comparing map lengths between populations.

### Distributions of COs along chromosomes

We investigated how COs vary along chromosomes in relation to their distance to the centromere and to the nearest telomere. Centromere locations were identified in all but three chromosomes (chromosomes 2, 4, and 8) by BLASTing the 186-bp threespine stickleback centromeric repeat motif (Cech and Peichel 2015) against the reference genome and taking the top hit for each chromosome. Each motif fell within an area of reduced nucleotide diversity, as expected of a centromere.

We also investigated whether male and female recombination rates are correlated along the chromosomes. We folded each chromosome in half, and measured CO locations as the relative distance from the nearest end. Next, we divided the folded chromosomes into *n* segments of equal size, where *n* varied from 2 to 20. We then calculated the relative map length of each segment by dividing the number of COs in each segment by the total number of COs on the chromosome. Finally, we performed least-squares linear regression in R v3.2.3 to test whether there was a significant correlation between relative male and female map lengths across these segments at each of the different scales. We used the same method to calculate the correlations between males and female relative map lengths at different scales along the long arm of each chromosome. We did not perform this analysis for the short arms because several of them did not recombine in any individual.

We used linear mixed models to test the effects of telomeres and centromeres on recombination rates. We divided each chromosome into 10 equal segments and used number of COs within each segment as the dependent variable, with separate analyses for each sex. Relative distances between the center of each segment and the nearest telomere was calculated by scaling by half the chromosome’s length. We assume that suppression of recombination around the centromere depends on absolute rather than relative distance. We therefore parameterized distance to the centromeres in Mb, and scaled by the maximum arm length to improve fitting of linear mixed models. (Models with relative distance from the centromere, in which distance to the centromere was scaled by the length of its chromosome, had uniformly lower support than models in which distances to the centromere were scaled by the maximum possible distance from a centromere, confirming the validity of this assumption.) The sex chromosomes (9 and 19) and the chromosomes that did not contain the centromeric repeat motif (2, 4, and 8) were excluded from this analysis. Models were constructed in R package lme4 v1.1-14 (Bates *et al.* 2015) with the distances to the nearest chromosome end and to the centromere as fixed effects, and the chromosome as a random effect. The best-fit model was identified from Akaike information criterion (AIC) values calculated in R v3.2.3. We also performed similar analyses for long arms only. (Distance to centromere and distance to chromosome end are perfectly correlated on short arms.)

We compared locations of all COs and genes using the approach of Smeds *et al.* (2016). Gene annotations were based on the annotations from Jones *et al.* (2012) translated into coordinates for the most recent threespine stickleback reference genome (Glazer *et al.* 2015). We tested for nonrandom associations between CO and gene locations using 10,000 simulations in which a set of CO intervals of the same lengths as those observed were randomly placed on each chromosome. We used the same approach to generate a null distribution for the expected number of COs falling within recombination hotspots (*i.e.*, windows of fixed length containing multiple COs).

### Interspecific genomic differentiation

To test whether recombination rates correlate with differentiation between species, we used VCFtools v1.15 (Danecek *et al.* 2011) to estimate *F*_ST_ (Weir and Cockerham 1984) between *G. nipponicus* and *G. aculeatus* for the high-quality SNPs used in our LDhelmet analysis (excluding the sex chromosomes, 9 and 19). Although reproductive barriers exist between these species (Kitano *et al.* 2007; Kitano *et al.* 2009), they occasionally hybridize; one of the wild-caught mothers in our crosses was an F_1_ hybrid, while other females exhibited varying degrees of hybrid ancestry. *F*_ST_ estimates were scaled by the mean *F*_ST_ for the whole chromosome to control for variation between chromosomes. We also calculated mean relative *F*_ST_ over the same segments used in our analyses of the distribution of COs along folded chromosomes and along long chromosome arms, and estimated the correlation between the two using R 3.2.3. Finally, we constructed linear mixed models using the R package lme4 v1.1-14 (Bates *et al.* 2015) with chromosome as a random effect to test whether *F*_ST_ (measured across 100 kb non-overlapping windows) is best predicted by the distance to the centromere, the relative distance to the chromosome center, or a combination of the two.

### Data availability

Sequence data that support the findings of this study have been deposited in NCBI’s Short Read Archive (http://www.ncbi.nlm.nih.gov/sra/SRP135745), reference number SRP135745.

## RESULTS

We identified 698 COs that occurred along the 21 stickleback chromosomes during 30 male meioses and 24 female meioses. COs were localized with a median resolution of 2.3 kb, and 79% of COs were located to within 10 kb (Figure S1 in File S1). Overall recombination rates are higher in females than males. Males averaged 0.48 COs per chromosome, consistent with one obligate CO per chromosome per meiosis. (COs affect only two of the four chromatids, so the total number of COs during meiosis is on average twice the number of COs observed in the gametes.) In contrast, females averaged 0.79 COs observed per chromosome per meiosis. The total recombination map lengths were 1007 cM in males and 1650 cM in females. The sex-averaged map length (1328 cM), is consistent with previous results (Roesti *et al.* (2013): 1251 cM; Glazer *et al.* (2015): 1570 cM and 1963 cM).

### Sex differences between chromosomes

Much of the difference between overall male and female recombination rates is due to a greater number of chromosomes with multiple COs in females. As discussed in the Methods, we are unable to assign crossovers to individual gametes, so we cannot determine if one crossover occurred in each gamete, or if a double crossover happened in one gamete and no crossover in the other (Figure 1B). The 59 cases in which three or more COs occurred on a single chromosome in a sibling pair were informative, however, since these imply that at least one of the gametes contained a double crossover. Of these, 85% occurred in females.

**Figure 2:**
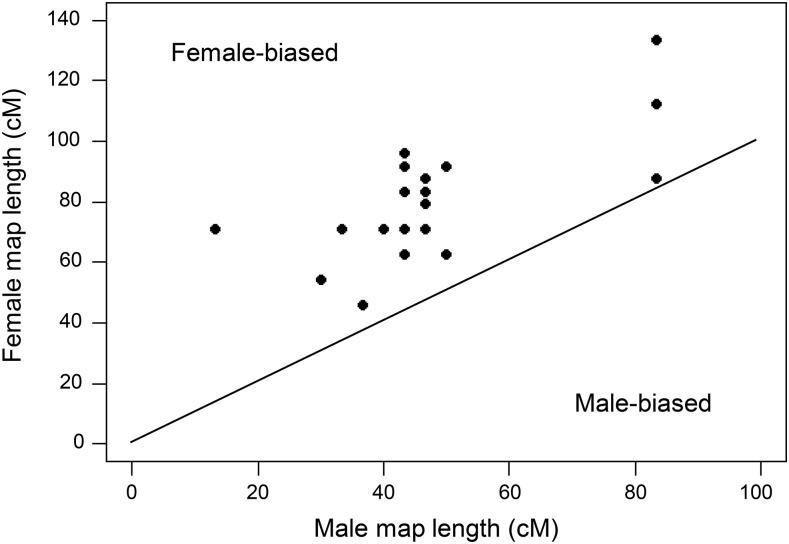
Male *vs.* female map lengths for the 21 stickleback chromosomes. All chromosomes exhibit female-biased recombination. The correlation is highly significant (*r* = 0.67, *P* < 10^−3^).

Map lengths for the 21 chromosomes are correlated in the two sexes, but are consistently higher in females (Figure 2). Ratios of the female to male recombination map lengths range from a low of 1.05 on chromosome 7 to a high of 5.31 on chromosome 18 (Table 1). The abnormally elevated ratio on chromosome 18 results because only four COs were observed in males. This may be an artifact of misassembly errors in the reference genome or phasing issues reflected in frequent shifts in allele sharing patterns between siblings at one end of that chromosome, both of which inhibit our ability to confidently detect COs. (Assuming one obligate CO occurs per chromosome, the probability of observing this few COs is *P* < 10^−4^.) No other chromosome has a ratio of female to male recombination map lengths greater than 2.21.

**Table 1:** Sex-specific recombination map lengths for each chromosome in *Gasterosteus* sticklebacks. Chromosome 19 is a sex chromosome in both *G. nipponicus* males and *G. aculeatus* females, while chromosome 9 is a neo-sex chromosome in G. nipponicus males.

STICKLEBACK CHROMOSOME	FEMALE RECOMBINATION MAP LENGTH	MALE RECOMBINATION MAP LENGTH	RATIO: FEMALE/MALE MAP LENGTH
1	112.5	83.3	1.35
2	95.8	43.3	2.21
3	62.5	50.0	1.25
4	133.3	83.3	1.60
5	70.8	46.7	1.52
6	70.8	40.0	1.77
7	87.5	83.3	1.05
8	62.5	43.3	1.44
9 (neo-sex)	83.3	46.7	1.78
10	70.8	33.3	2.13
11	62.5	50.0	1.25
12	91.7	50.0	1.83
13	79.2	46.7	1.70
14	54.2	30.0	1.81
15	45.8	36.7	1.25
16	83.3	43.3	1.92
17	87.5	46.7	1.88
18	70.8	13.3	5.31
19 (sex)	91.7	43.3	2.11
20	70.8	43.3	1.63
21	62.5	50.0	1.25
**Total**	**1650.0**	**1006.7**	**1.64**

**Figure 3:**
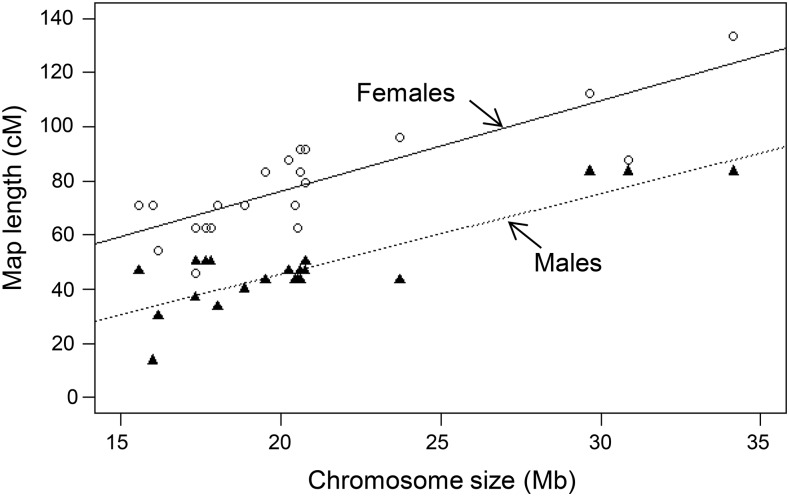
Recombination map length correlates with physical size of chromosomes in both males (filled triangles) and females (hollow circles). The least-squares linear regressions for each sex are highly significant (*P* < 10^−5^).

As seen in Figure 3, a chromosome’s map length is highly correlated with its physical length in both males (*r* = 0.87) and females (*r* = 0.84). However, recombination rates, measured as cM/Mb, are not significantly correlated with chromosome size in either sex (males: *r* = 0.097, *P* = 0.29; females: *r* = 0.13, *P* = 0.56; linear regression chosen because it best fits data). Likewise, recombination rates in males and females are not significantly correlated between chromosomes (*r* = 0.16, *P* = 0.49) (Figure S2 in File S1).

Females show higher variation between individuals in CO count, even after scaling for differences in means. The coefficient of variation for the number of COs in females in 0.20, compared to 0.16 for males. Although this difference is not statistically significant (*P* = 0.39, Feltz and Miller (1996)), it is consistent with the observation that multiple crossovers on a chromosome are more frequent in females.

### Sex differences along chromosomes

**Figure 4:**
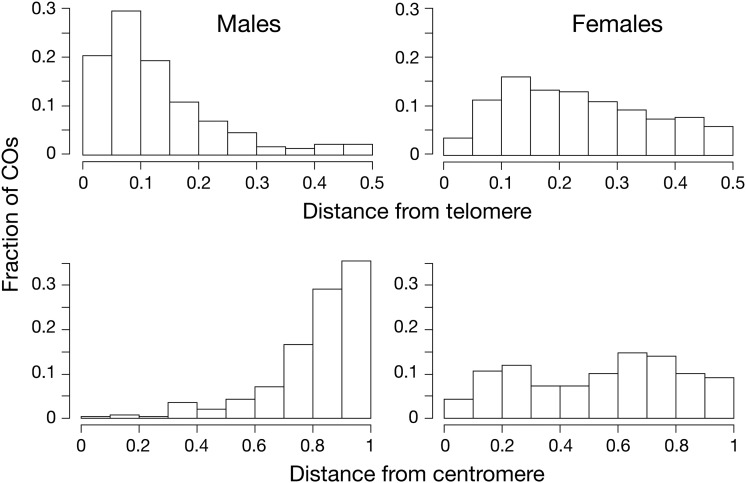
Distribution of crossovers along the chromosomes in sticklebacks. Top: Relationship between recombination rate and relative distance from nearest telomere, scaled by the chromosome length. Data for males are based on 30 meioses and 302 COs; data for females are based on 24 meioses and 396 COs. Bottom: Relationship between recombination rate and relative distance from centromere. Chromosomes 2, 4, and 8 were excluded because centromere location could not be accurately determined. This reduces the number of COs to 251 in males and 326 in females. The differences between sexes are highly significant for both measures (two-sample Kolmogorov-Smirnov test: *P* < 10^−15^).

We first asked how the frequency of COs varies with distance from the nearest telomere. Sex-averaged recombination rates are elevated toward the ends of chromosomes, as previously observed by Roesti *et al.* (2013) and Glazer *et al.* (2015). However, as shown in Figure 4, the distribution of COs along each chromosome differs dramatically between the sexes. In males, COs are overwhelmingly clustered toward the end of chromosomes. In females, however, COs are distributed much more uniformly.

We next asked whether the frequency of COs varies with the distance from the centromere. Figure 4 shows that the effect of the centromere on the long arms is dramatically different in the two sexes. In males, almost no COs occurred near the centromere. In females, recombination is reduced in the regions immediately adjacent to the centromere, but to much less of an extent than in males.

**Figure 5:**
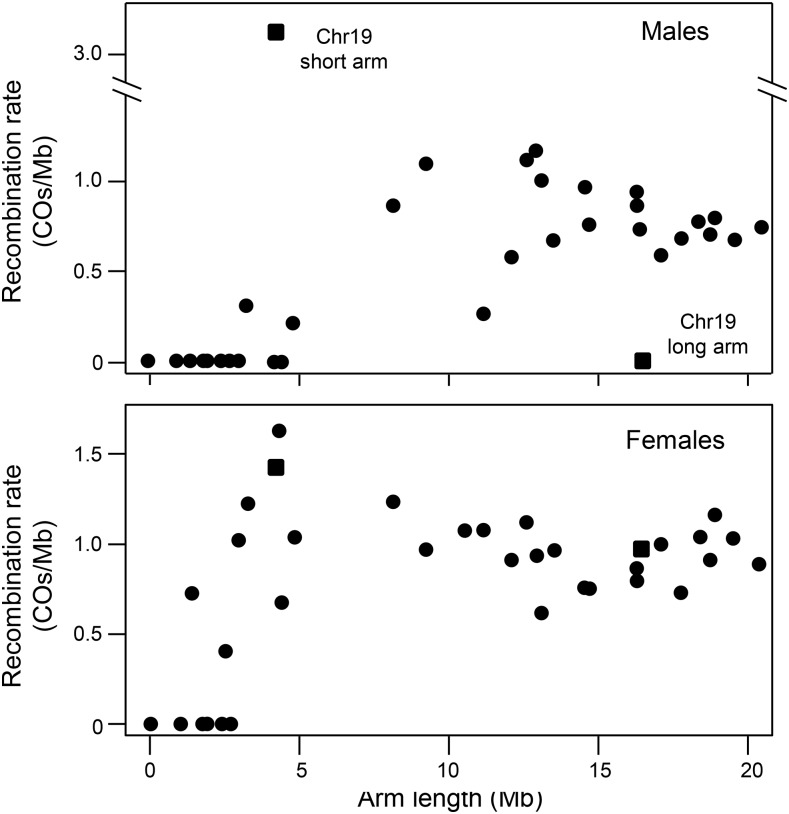
Recombination rates (COs/Mb) for each chromosome arm as a function of their length. Top: In males, recombination is highly suppressed on short arms (less than 7 Mb long) and completely suppressed on very short arms (less than 4 Mb long), likely due to proximity to the centromere. The notable exception is the sex chromosome (chromosome 19), where all COs occur on the short arm, which is the pseudoautosomal region, and none on the long arm. Bottom: In females, normal recombination rates occur on some arms as short as 2 Mb long.

We next investigated whether the suppression of recombination near centromeres in males affects which arm of a chromosome is most likely to recombine. Figure 5 shows that recombination is dramatically suppressed on short chromosome arms when they are less than 5 Mb long. The only exception is chromosome 19, which is the sex chromosome in both species. Here all COs in males fell within a pseudoautosomal region (PAR) on the short arm of the chromosome, as previously observed by Ross and Peichel (2008). Among the 12 autosomes in which the short arm comprises less than 30% of the chromosome, only one CO occurred on the short arm in a male (*χ*^2^ = 19.5, *P* = 10^−5^). These results suggest that the suppression of recombination near the centromere in males effectively inhibits nearly all recombination on the short arms of acrocentric chromosomes. In females, reduced recombination on short chromosome arms is also observed, but to a much lesser extent.

To assess the relative effects of telomeres and centromeres on CO rates, we constructed linear mixed models of crossover frequency that include both distance from centromere and relative distance from the nearest telomere as fixed effects. In males, the best-supported models for entire chromosomes and for long chromosome arms include both telomere and centromere effects (ΔAIC = 33.6 from the next highest supported model for whole chromosomes, ΔAIC = 31.1 for long chromosome arms), with distance from the centromere as the more important factor (Tables S1 and S2 in File S1). In females, the distribution of COs along entire chromosomes is best described by a model that includes the centromere effect but not the telomere effect (Table S1 in File S1). On long arms, the best supported model in females includes the telomere effect, but not the centromere effect (Table S2 in File S1). However, neither of these models for females are appreciably better than alternative models, including the null model with no telomere or centromere effect (ΔAIC = 5.9 from the null model for whole chromosomes, ΔAIC = 6.4 for long chromosome arms).

We find positive correlations between males and females in the distribution of COs at different spatial scales. This relationship holds across all scales tested, ranging from 2 to 20 segments per chromosome half (Figure S3 in File S1). Correlations are strongest for large segments (when chromosome halves are divided into halves and into thirds), consistent with broad differences in recombination rates between chromosome ends and centers. Similar results hold for the distribution of COs along the long arms (Figure S4 in File S1).

### Recombination hotspots

We defined hotspots as 100 kb regions containing multiple COs (following Smeds *et al.* (2016). When both sexes were pooled, 43% of all COs fell within hotspots, significantly more than expected by chance (median expected = 30%, *P* < 10^−5^). In males, 45% of COs fell within hotspots (median expected = 15%, *P* < 10^−5^), but in females, only 23% of COs fell within hotspots (median expected = 18%, *P* = 0.015). Next, we used a stricter criterion that defined hotspots as 10 kb regions containing multiple COs. When both sexes were pooled, 14% of COs fell within hotspots (median expected = 7%, *P* < 10^−5^). In males 17% of COs fell within hotspots (median expected = 4%, *P* < 10^−5^), while in females 7% of COs fell within hotspots (median expected = 4%, *P =* 0.013).

The fraction of CO intervals that overlap genes is not significantly different from random expectations (Figure S5 in File S1). Likewise, the difference between the sexes is not statistically significant (χ^2^ = 0.13, *P =* 0.72). Finally, the overlaps of CO intervals with different categories of gene regions, such as exons, introns, and promoters (defined as any region 2 kb upstream from the transcription start sites) are not significant (Figure S5 in File S1).

Crossovers are associated with slightly enriched GC content in both sexes. CO intervals had 48.1% GC content in males and 46.7% GC content in females, compared to the genome-wide average of 44.6%. (Differences between all three comparisons are significant at *P* < 2 × 10^−4^.) Enrichment is expected in males because GC content is elevated near telomeres where male COs cluster. The mean GC content for the two distal quartiles of each chromosome was 45.2%, while the mean GC for the two central quartiles of each chromosome was 43.7%. To determine if recombination is correlated with GC content on finer scales, we calculated GC content within each CO interval and compared it to its two 250 kb flanking regions. GC content is significantly greater in CO intervals than in the flanking regions for both males and females (males: 48.1% *vs.* 46.5%; females: 46.7% *vs.* 45.2%; *P* < 10^−6^ for each sex by paired *t*-tests).

### Recombination on the sex chromosomes

Sex chromosomes typically exhibit recombination patterns that differ from autosomes. In *G. aculeatus*, sex is determined by chromosome 19 (Peichel *et al.* 2004). In *G. nipponicus*, however, the ancestral Y chromosome fused with chromosome 9 to form a neo-Y. The homolog of the neo-Y remains unfused but segregates as a neo-X (Kitano *et al.* 2009). Recombination between the ancestral X and Y (that is, chromosome 19) was restricted to the first 15% (2.9 Mb) of the chromosome that is distal from the fusion, consistent with previous studies (Ross and Peichel 2008; Roesti *et al.* 2013). This pseudoautosomal region falls on the short arm of chromosome 19 (see also Roesti *et al.* 2013; White *et al.* 2015). This pattern of recombination contrasts strongly with that on the autosomes, where no recombination occurs on the short arms of chromosomes with similar long-to-short arm ratios (Figure 5). A second pseudoautosomal region between the neo-X and neo-Y occurs on the half of chromosome 9 that is distal to the fusion with chromosome 19, consistent with Natri *et al.* (2013). The large majority (79%) of COs that occurred in this region fell within the distal 15% (3.1 Mb) of the chromosome.

The fusion in *G. nipponicus* has not had a substantial effect on overall recombination rates for either chromosome 9 or 19. Both average approximately one crossover per meiosis in males, even though they comprise a single neo-Y chromosome. One crossover on each end of the neo-Y may be required for proper synapsis during meiosis because the ancestral X and neo-X remain unfused.

### Population historic recombination rates

To compliment and verify results from the experimental crosses, we estimated the sex-averaged historic recombination rates along each chromosome for each species using LDhelmet v.1.9 (Chan *et al.* 2012). The results show the same general patterns seen in our crosses (Figure S6 in File S1). Recombination patterns along the genome were strongly correlated between the two species of sticklebacks (*r* = 0.83 across 1 Mb sliding windows), excluding chromosome 9 where recombination has been altered by its recruitment as a neo-sex chromosome in *G. nipponicus* (Kitano *et al.* 2009; Yoshida *et al.* 2014).

### Recombination rate and interspecific differentiation

Threespine stickleback ecotypes show elevated differentiation toward the centers of chromosomes, and it is thought this pattern results from lower sex-averaged recombination rates in these centers (Roesti *et al.* 2012; Berner and Roesti 2017; Samuk *et al.* 2017). For example, lack of recombination facilitates selection against blocs of linked weakly deleterious foreign alleles (Butlin 2005). Selection that causes population differences in allele frequencies at one locus also has a stronger effect on the allele frequencies of nearby loci in regions of low recombination, resulting in “islands” of genomic differentiation unrelated to speciation (Noor and Bennett 2009; Cruickshank and Hahn 2014). We asked whether recombination is also correlated with differentiation between threespine and Japan Sea sticklebacks. We measured differentiation by calculating $F_{\text{ST}}^{*}$, a version of *F*_ST_ that is scaled by the chromosome’s mean *F*_ST_, in 100 kb windows.

**Figure 6:**
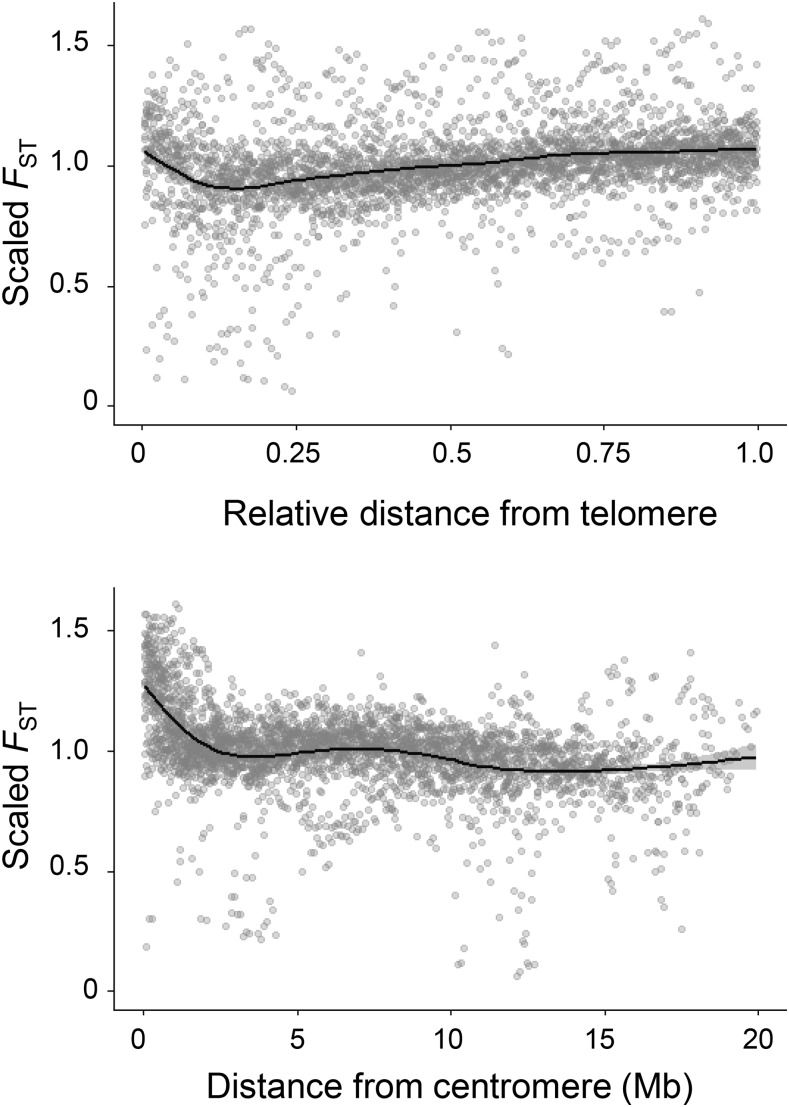
Interspecific differentiation between *G. aculeatus* and *G. nipponicus* as function of relative distance from chromosome center (top) and raw distance from centromere (bottom). Data points represent mean *F*_ST_ in 100 kb windows, scaled by the mean *F*_ST_ for the whole chromosome. Distance from chromosome center is scaled by chromosome length.

We find that differentiation between *G. nipponicus* and *G. aculeatus* has a strong negative correlation with the sex-averaged recombination rate along the chromosomes (*r* = -0.85, *P* = 0.002). This correlation is higher than the correlations for male or female recombination considered separately (*r* = -0.69, *P* = 0.03 and *r* = -0.68, *P* = 0.03, respectively). The ratio of F_ST_* on short chromosome arms to F_ST_* on long chromosome arms is negatively correlated with the relative length of the short arm, in accordance with our finding that the centromere reduces recombination on the short arm of acrocentric chromosomes (Figure S7 in File S1). As with recombination, F_ST_* is best predicted by a linear model that includes both distance to telomere as well as distance to centromere, with larger loadings for the centromere effect (ΔAIC = 112 from the next highest supported model) (Table S2 in File S1). This results in low differentiation toward chromosome ends (increasingly slightly at the telomeres due to low recombination rates in females) and greatest differentiation near the centromere (Figure 6, Figure S6 in File S1).

## DISCUSSION

These results provide the first analysis of sex-specific differences in recombination in *Gasterosteus* sticklebacks. We demonstrate female-biased heterochiasmy across all chromosomes, with an overall map length 1.64 times longer in females than males. The degree of sex bias in threespine sticklebacks is similar to that in ninespine sticklebacks (*Pungitius pungitius*), where map lengths are between 1.5 and 1.9 times longer in females (Rastas *et al.* 2015).

We also demonstrate for the first time that distributions of COs along the chromosomes differ strikingly between male and female sticklebacks. Recombination rates in males are influenced both by a telomere effect, in which the vast majority of COs occur at chromosome ends, as well as a centromere effect, in which recombination is reduced within approximately 5 Mb of the centromere. In contrast, recombination rates in females are much more uniform along the chromosomes, with weak evidence for any centromere or telomere effects. Although a pattern of increased recombination at chromosome ends and decreased recombination at the centromere has been identified in many other species (*e.g.*, Devaux *et al.* 1995; Broman *et al.* 1998; Chen *et al.* 2002; Woram *et al.* 2004; Shifman *et al.* 2006; Moen *et al.* 2008; Wong *et al.* 2010; Jones *et al.* 2013; Brelsford *et al.* 2015; Ma *et al.* 2015; Smeds *et al.* 2016), our study presents the first quantitative analysis of the relative importance of the telomere and centromere effects in driving recombination differences between sexes.

Stickleback ecotypes show elevated differentiation toward the centers of chromosomes, and it is thought this pattern results from the lower sex-averaged recombination rates there (Roesti *et al.* 2012; Berner and Roesti 2017; Samuk *et al.* 2017). Our results show that this “chromosome center biased differentiation” is driven both by the high frequency of COs near telomeres, as well as a positive effect of centromere location on differentiation that is independent of, and stronger than, the telomere effect. Both patterns are products of the strong effect of telomeres and centromeres on recombination in males but not females, indicating the importance of sex-specific recombination patterns for evolution.

Below, we compare these results with those from other taxa, discuss hypotheses for the mechanistic and evolutionary basis of sex differences in recombination, and consider the potential evolutionary consequences of these differences.

### Heterochiasmy

Female-biased heterochiasmy, as observed in sticklebacks, is the most common pattern in animals (Brandvain and Coop 2012). Our data suggests that heterochiasmy in sticklebacks results primarily from a higher frequency of chromosomes with multiple COs in females. This pattern may result from sexual dimorphism in crossover interference, which is known to be stronger in males than females in humans (Hou *et al.* 2013). Our experimental design prevents us from quantifying crossover interference because (as discussed earlier) we cannot distinguish a double crossover in one gamete from two single crossovers in the gametes that contributed to siblings. However, the vast majority of instances of multiple COs on a single chromosome that we could identify occurred in females. In contrast, males average approximately one CO per chromosome. This outcome is expected if one CO per chromosome (but not per chromosome arm) is required for successful pairing during meiosis, as observed in other taxa (Mather 1938; Jones 1983; Martini *et al.* 2006; Dumont 2017), including sticklebacks Roesti *et al.* (2013). Females also show greater variance between individuals in crossover count than males, even after accounting for differences in the means. A similar pattern is seen in humans (Ottolini *et al.* 2015).

### Sex differences along chromosomes

The sexual dimorphism in the distributions of COs along chromosomes seen in sticklebacks (Figure 4) is also found in almost all other species. Male-biased recombination near telomeres has been observed in humans (Broman *et al.* 1998), most other mammals (Shifman *et al.* 2006; Wong *et al.* 2010; Ma *et al.* 2015), birds (Smeds *et al.* 2016), amphibians (Brelsford *et al.* 2015; Brelsford *et al.* 2016), teleost fishes (Singer *et al.* 2002; Gharbi *et al.* 2006; Moen *et al.* 2008; Rexroad *et al.* 2008; CastañO-Sánchez *et al.* 2010), mollusks (Jones *et al.* 2013; Wang *et al.* 2016), and plants (Devaux *et al.* 1995; Lagercrantz and Lydiate 1995; Giraut *et al.* 2011). Similarly, centromeres commonly suppress recombination (Lambie and Roeder 1988; Chen *et al.* 2002; Ellermeier *et al.* 2010), with a large male-specific centromere effect observed in humans (Broman *et al.* 1998) and teleost fishes (Woram *et al.* 2004; Moen *et al.* 2008). The consistency of these patterns across phyla makes a null model in which selection acts solely on the sex-averaged recombination rate and sex-specific patterns evolve via neutral genetic drift implausible. Exceptions to the general pattern do occur, however, indicating that the evolution of sex-specific recombination is not constrained by the mechanics of meiosis. Recombination rates are highly elevated near telomeres in both sexes of the domestic pig (*Sus scrofa*) (Tortereau *et al.* 2012) and zebra finch (*Taeniopygia guttata*) (Backström *et al.* 2010). Heterochiasmy is strongly male-biased and female recombination rates are strongly reduced near centromeres in opossums (*Monodelphis domestica*) (Samollow *et al.* 2007). Finally, broad differences between the sexes in the distribution of COs varies between chromosomes in the sequentially hermaphroditic gilthead sea bream (*Sparus aurata*) (Franch *et al.* 2006).

Female-biased heterochiasmy may be a product of sex differences in the distribution of COs along chromosomes (Petkov *et al.* 2007). Since COs in males are strongly clustered near telomeres, crossover interference from one CO at the end of the chromosome may prevent a second CO from occurring on that chromosome arm. In contrast, recombination in females is relatively uniform along the chromosomes, which allows more opportunities for two crossovers to occur on the same arm. Male-biased recombination in telomeres does not necessarily entail female-biased heterochiasmy, however, as it is also present in species with male-biased heterochiasmy, including Japanese flounders (*Paralichthys olivaceus*), domestic cattle (*Bos taurus*), and collared flycatchers (*Ficedula albicollis*) (Castaño-Sánchez *et al.* 2010; Ma *et al.* 2015; Smeds *et al.* 2016). These species may lack CO interference, or CO interference may suppress recombination across entire chromosomes, thereby minimizing the effects of CO clustering on heterochiasmy.

### Fine-scale patterns of recombination

Heterochiasmy can result from sex-specific recombination hotspots. We found evidence for recombination hotspots in both sexes. However, they primarily result from clustering of COs in broad regions, rather than fine-scale chromosome structure. Hotspots are most evident in males, where COs are concentrated near the ends of chromosomes. Evidence for hotspots is much weaker in females.

Hotspots in birds, yeast, and plants are typically located near transcription start sites, likely because the open chromatin regions that favor transcription also favor the double stranded breaks that initiate COs (Pan *et al.* 2011; Drouaud *et al.* 2013; Hellsten *et al.* 2013; Smeds *et al.* 2016). By contrast, in humans and many other mammals, recombination rates are reduced near transcription start sites and higher in intergenic regions, due primarily to the distribution of binding sites for the zinc finger protein PRDM9 (Myers *et al.* 2005; Coop *et al.* 2008; Baudat *et al.* 2010; Berg *et al.* 2010; Parvanov *et al.* 2010; Brick *et al.* 2012; Auton *et al.* 2013). Sticklebacks have lost PRDM9α, the homolog of PRDM9 responsible for mediating recombination in teleost fishes (Baker *et al.* 2017), as we found no evidence for any paralog of PRDM9 in the stickleback reference genome other than the single previously annotated copy (ENSGACG00000003343). The structure of this protein, including the arrangement of the zinc finger array and the lack of SSXRD and KRAB domains, is characteristic of PRDM9β, which does not affect recombination in *Xiphophorus* fish (Baker *et al.* 2017). Due to this loss of PRDM9α in sticklebacks, we expected COs to cluster near transcription start sites. We found no evidence, however, for either enrichment or reduction of COs near genes, transcription start sites, or promoter regions. It is possible that recombination in sticklebacks is unrelated to fine-scale variation in gene content, a situation also found in honeybees (*Apis mellifera*), worms (*Caenorhabditis elegans*), and fission yeast (*Schizosaccharomyces pombe*) (Cervantes *et al.* 2000; Kaur and Rockman 2014; Wallberg *et al.* 2015). Alternatively, we may have failed to detect fine-scale patterns because of the relatively small number of COs in our data set.

GC content is elevated in intervals containing COs in both males and females. Positive correlations between recombination rates and GC content are present in many species (including humans, birds, and yeast), and are believed to result from GC-biased gene conversion during the repair of recombination-initiating double stranded breaks (Galtier *et al.* 2001; Lesecque *et al.* 2013; Arbeithuber *et al.* 2015; Smeds *et al.* 2016). GC content is also significantly higher in male CO intervals than in females. These patterns are driven in part by the concentration of male COs in chromosome ends, which have higher GC content. However, GC content is also elevated within CO intervals compared to local flanking regions. Sexual dimorphism in the correlation of recombination with GC content is also found in other taxa, but the direction and magnitude of the dimorphism varies. In humans and pigs, GC content is more highly correlated with female recombination rate (Tortereau *et al.* 2012; Bhérer *et al.* 2017), while in dogs (which lack PRDM9), GC content is positively correlated with male but not female recombination rates (Wong *et al.* 2010).

### Genomic differentiation between species

Our finding that recombination rate is strongly correlated with genomic differentiation between *G. aculeatus* and *G. nipponicus* matches theory as well as observations from several other species, including humans (*e.g.*, Hellmann *et al.* 2003; Kulathinal *et al.* 2008; Keinan and Reich 2010; Cruickshank and Hahn 2014; Burri *et al.* 2015; Vijay *et al.* 2017). This relationship has previously been observed between ecotypes of threespine sticklebacks, which exhibit elevated differentiation in the centers of most chromosomes (Roesti *et al.* 2012; Roesti *et al.* 2013; Samuk *et al.* 2017). Roesti *et al.* (2012) suggested that this pattern, which they term “chromosome center biased differentiation”, is independent of centromere location.

We found that differentiation along chromosomes is best predicted by two independent effects. The strongest is a centromere effect in which reduced recombination drives elevated differentiation around the centromere. The second predictor is a telomere effect, in which elevated recombination near the telomeres causes relative differentiation to increase when moving away from the ends of chromosomes toward the center. The relative distances to the telomere and the chromosome center are perfectly correlated, and so we cannot distinguish mechanistically whether the telomere or chromosome center is driving this pattern. The distinction, however, is largely semantic: elevated relative differentiation at the center implies reduced relative differentiation at the telomeres, and vice versa. The telomere effect acts independently of centromere location, and could explain why chromosome center biased differentiation is observed in organisms with holocentric chromosomes, such as *C. elegans* worms and *Heliconius* butterflies (Berner and Roesti 2017). In metacentric chromosomes, both the centromere and telomere effects combine to increase differentiation near the chromosome center. In acrocentric chromosomes, the highest differentiation occurs at the centromere, but the telomere effect also increases differentiation at the chromosome center. Both the centromere and telomere effects in sticklebacks are driven primarily by recombination patterns in males, reinforcing the importance of sex-specific recombination rates for evolution.

There are two possible explanations for the discrepancy between our results and previous studies relating centromere location, recombination rate, and genomic differentiation in sticklebacks. First, we identified centromere locations by BLASTing the recently-identified centromeric repeat motif (Cech and Peichel 2015) to the stickleback reference genome, whereas Roesti *et al.* (2012) used relative arm lengths measured from chromosome spreads (Urton *et al.* 2011) to estimate centromere location. These two approaches give inconsistent results for some chromosomes, particularly acrocentric chromosomes where we expect great uncertainty in the ratios of short to long arm lengths measured from karyotype data. Centromere locations identified using our method are more highly associated with genomic features that are typical of centromeric regions, such as reduced heterozygosity and greater differentiation between species, than locations estimated from karyotype data.

Second, although hybridization occurs between threespine and Japan Sea stickleback, there are strong reproductive barriers between them (Kitano *et al.* 2007). As a result, gene flow between these two species is likely much less than gene flow between sympatric ecotypes of threespine stickleback. Berner and Roesti (2017) and Samuk *et al.* (2017) hypothesize that chromosome center biased differentiation between stickleback ecotypes is predominantly driven by selection against introgression of foreign alleles at locally-adapted loci. In contrast, linked selection that reduces intrapopulation diversity in regions of low recombination may be a more important driver of genomic differentiation between *G. aculeatu*s and *G. nipponicus* than resistance against introgression (Noor and Bennett 2009; Cruickshank and Hahn 2014).

### Potential sources of error

There are three potential biases resulting from our method for detecting COs. First, as discussed in the Methods, we are unable to confidently detect COs that occur close to the ends of chromosomes, or to distinguish independent COs in sibling gametes that are separated by less than 400 kb. Failure to detect COs is expected to be more common in males, where COs are concentrated toward chromosome ends. This, in turn, will overestimate female-biased heterochiasmy and underestimate the degree of sexual dimorphism in CO distribution.

Second, chromosomal rearrangements (*e.g.*, inversions or transpositions) that differ from the reference genome can result in allele-sharing patterns at the rearrangement breakpoints that mimic those for COs. This will overestimate the number of COs, and reduce our power to detect fine-scale patterns affecting local recombination rates. Rearrangements shared by multiple individuals can also lead to false detection of recombination hotspots. The *G. aculeatus* females in our crosses are less diverged from the *G. aculeatus* reference genome (Glazer *et al.* 2015) than are the *G. nipponicus* males. Consequently, issues arising from chromosomal rearrangements are more likely to affect results for males, which will lead to an underestimate of female-biased heterochiasmy.

Third, our hybrid cross design confounds sex and species differences in recombination, and we cannot rule out the possibility that the observed differences in males and females are species-specific. There are, however, several reasons to believe that differences between the sexes are likely much larger than those between the species. First, our sex-averaged map is consistent with sex-averaged maps previous developed for *G. aculeatus* (Roesti *et al.* 2013; Glazer *et al.* 2015). It is also consistent with maps for *Pungitius pungitius* (Rastas *et al.* 2015), whose most recent common ancestor with *Gasterosteus* sticklebacks is about seven times older than the ancestor of threespine and Japan Sea sticklebacks. This similarity suggests that recombination patterns are relatively conserved across stickleback species. Second, our estimates of historical sex-averaged recombination patterns are strongly correlated between species, as well as with data from our experimental crosses. Third, patterns of sex-specific differences in recombination seen in our crosses are consistent with patterns seen in many other species. Together, these lines of evidence indicate that broad-scale differences in recombination between males and females in our experimental crosses likely reflect sex differences rather than species differences.

### Causes and consequences of sex differences in recombination

Several evolutionary hypotheses have been proposed to explain sex differences in recombination (reviewed in Brandvain and Coop (2012)). Differences in how selection acts on males and females will generally not favor the evolution of sex differences in recombination (Lenormand 2003). Selection on male and female gametes, however, can do so (Lenormand and Dutheil 2005). Selection against aneuploidy typically will favor telomere-biased recombination, as chiasmata occurring close to the centromere are frequently associated with improper segregation during meiosis (May *et al.* 1990; Lynn *et al.* 2004; Lamb *et al.* 2005; Rockmill *et al.* 2006). Models developed by Brandvain and Coop (2012) show that meiotic drive can favor elevated recombination near centromeres in females despite the increased risk of aneuploidy. Selection on male recombination rates, however, is unaffected by female meiotic drive, favoring differences in recombination between the sexes. Our demonstration that recombination is strongly suppressed near the centromere in males, but not females, provides some of the best supporting evidence to date for the predictions of the meiotic drive model.

Sticklebacks are one of many taxa that show rapid turnover of sex chromosomes, and sex differences in recombination will have important consequences for this process (van Doorn and Kirkpatrick 2007; Ross *et al.* 2009; van Doorn and kirkpatrick 2010; Beukeboom and Perrin 2014). Recombination between sex chromosomes is governed by sex-specific recombination rates: recombination between X chromosomes is determined by the female rate, while recombination between the X and Y is determined by the male rate. The gain of sex linkage will immediately alter the recombination landscape of an entire chromosome. Consider a new XY system that arises via a new male-determining factor located near the center of a former autosome. The paucity of crossovers in chromosome centers in males will immediately cause partial or complete sex linkage of a large region of the chromosome. This sex-linkage effect could in fact contribute to sex chromosome turnover, which can be favored when a locus under sexually antagonistic selection is tightly linked to a novel sex-determining factor (Charlesworth and Charlesworth 1980; van Doorn and Kirkpatrick 2007; van Doorn and Kirkpatrick 2010). The analogous effect will not occur in turnovers leading to a new ZW system, however, because recombination rates in females are relatively uniform along chromosomes. This difference between XY and ZW systems could make transitions to new XY systems more frequent, and cause XY to be more common than ZW sex determination (van Doorn and Kirkpatrick 2007; van Doorn and Kirkpatrick 2010). Finally, once sex chromosomes are established, differences in recombination rates between them and autosomes can have major impacts on the evolution of sexually antagonistic genes and their distribution in the genome (Connallon and Clark 2010).

The extent to which sex differences in recombination influence sex chromosome turnover remains unexplored. We note, however, that evolutionary consequences of sex-specific recombination patterns are likely to be strongest in young sex chromosomes. Selection for reduced recombination between sex chromosomes can result in recombination patterns on sex chromosomes that differ fundamentally from typical sex-specific patterns. For example, all recombination between the stickleback X and Y occurs on the short arm of chromosome 19, in marked contrast to autosomes with similar arm length ratios, where most recombination occurs at the end of the long arm. Thus, studies of recently-formed sex chromosomes are most likely to provide insight into the potential evolutionary consequences of sex-specific differences in recombination.

## Supplementary Material

Supplemental Material is available online at www.g3journal.org/lookup/suppl/doi:10.1534/g3.118.200166/-/DC1.
