# Supplementary material for: Sex Differences in Recombination in Sticklebacks
Source: G3 (Bethesda). 2018 Apr 9;8(6):1971–83. doi: 10.1534/g3.118.200166 (PMC5982825; doi:10.1534/g3.118.200166)
Supplement: Supplementary file 1 [file 1971FileS1.pdf]

SUPPLEMENTAL MATERIAL

Supp. Table 1: Performance of linear mixed models for whole chromosomes. For recombination models, chromosomes were divided into 10 equal-length segments with total number of COs in each segment as the dependent variable. For differentiation, mean F_{ST} over 100 kb windows scaled by mean F_{ST} for the whole chromosome was the dependent variable. *Telomere* = distance from midpoint of segment to nearest chromosome end, scaled by half the chromosome length. *Centromere* = distance from midpoint of segment to the centromere, scaled by largest arm length across all chromosomes. *Chromosome* = random effect. Best supported models are those with the lowest Akaike information criterion (AIC) values.

MODEL	LOADINGS (t values)	AIC
**Male Recombination**		
Telomere + Centromere + Chromosome	*Telomere:* -3.0 (-6.3)<br>*Centromere:* 5.6 (10.1)<br>*Intercept:* -2.0 (-6.7)	628.4
Centromere + Chromosome	*Centromere*: 6.3 (10.4)<br>*Intercept:* -0.8 (-3.0)	662.0
Telomere + Chromosome	*Telomere:* -4.0 (-6.7)<br>*Intercept:* -0.6 (-1.8)	704.8
Chromosome	*Intercept:* 1.4 (7.3)	742.4
**Female Recombination**		
Centromere + Chromosome	*Centromere*: 1.3 (2.8)<br>*Intercept:* 1.3 (6.6)	579.4
Telomere + Centromere + Chromosome	*Telomere:* -0.4 (-1.0)<br>*Centromere:* 1.2 (2.6)<br>*Intercept:* 1.2 (4.4)	580.5
Telomere + Chromosome	*Telomere:* -0.6 (-1.5)<br>*Intercept:* 1.5 (6.2)	585.1
Chromosome	*Intercept:* 1.8 (15.2)	585.3
**Scaled Interspecific Differentiation (F_{ST}^*)**		
Telomere + Centromere + Chromosome	*Telomere:* 0.1 (10.8)<br>*Centromere:* -0.2 (-18.1)<br>*Intercept:* 1.1 (149.2)	-2560.1
Centromere + Chromosome	*Centromere*: -0.3 (-20.9)<br>*Intercept:* 1.1 (161.7)	-2448.3
Telomere + Chromosome	*Telomere:* 0.2 (14.8)<br>*Intercept:* 1.5 (162.4)	-2252.9
Chromosome	*Intercept:* 1.0 (242.5)	-2044.7

Supp. Table 2: Performance of linear mixed models for predicting male and female recombination along long arms of chromosomes. Arms were divided into 10 equal length segments with total number of COs in each segment as the dependent variable. *Telomere* = distance from mid-point of segment to the nearest end of chromosome, scaled by total chromosome length. *Centromere* = distance from mid-point of segment to the centromere, scaled by largest arm length across all chromosomes. *Chromosome* = random effect. Best supported models are those with the lowest Akaike information criterion (AIC) values.

MODEL	LOADINGS (t values)	AIC
**Male Recombination**		
Telomere + Centromere + Chromosome	*Telomere:* -2.5 (-5.6)<br>*Centromere:* 4.1 (8.2)<br>*Intercept:* -1.5 (-6.8)	553.8
Centromere + Chromosome	*Centromere*: 4.5 (11.2)<br>*Intercept:* -1.1 (-4.6)	584.9
Telomere + Chromosome	*Telomere:* -4.2 (-9.4)<br>*Intercept:* -0.6 (-2.8)	607.5
Chromosome	*Intercept:* 1.2 (7.7)	675.6
**Female Recombination**		
Telomere + Chromosome	*Telomere:* -1.1 (-2.9)<br>*Intercept:* 1.0 (5.1)	554.2
Telomere + Centromere + Chromosome	*Telomere:* -0.9 (-2.1)<br>*Centromere:* 0.5 (0.9)<br>*Intercept:* 0.9 (4.1)	555.3
Centromere + Chromosome	*Centromere*: 0.7 (1.8)<br>*Intercept:* 1.2 (5.4)	559.5
Chromosome	*Intercept:* 1.5 (13.9)	560.6

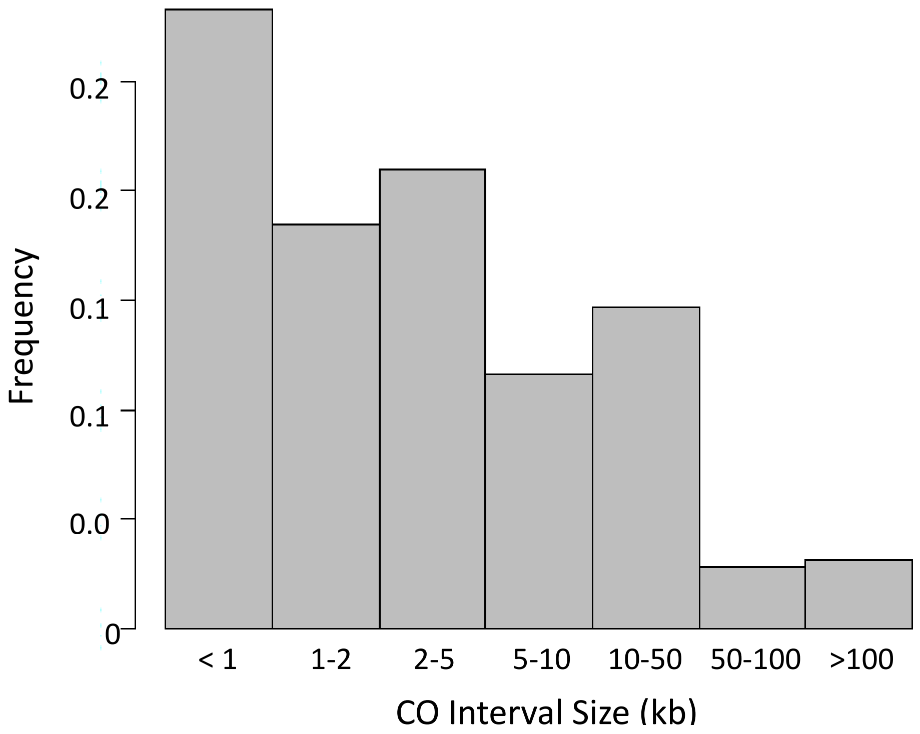

Supp. Fig. 1 – Resolution of crossover locations. Interval size is defined as the distance between adjacent SNPs bounding each crossover.

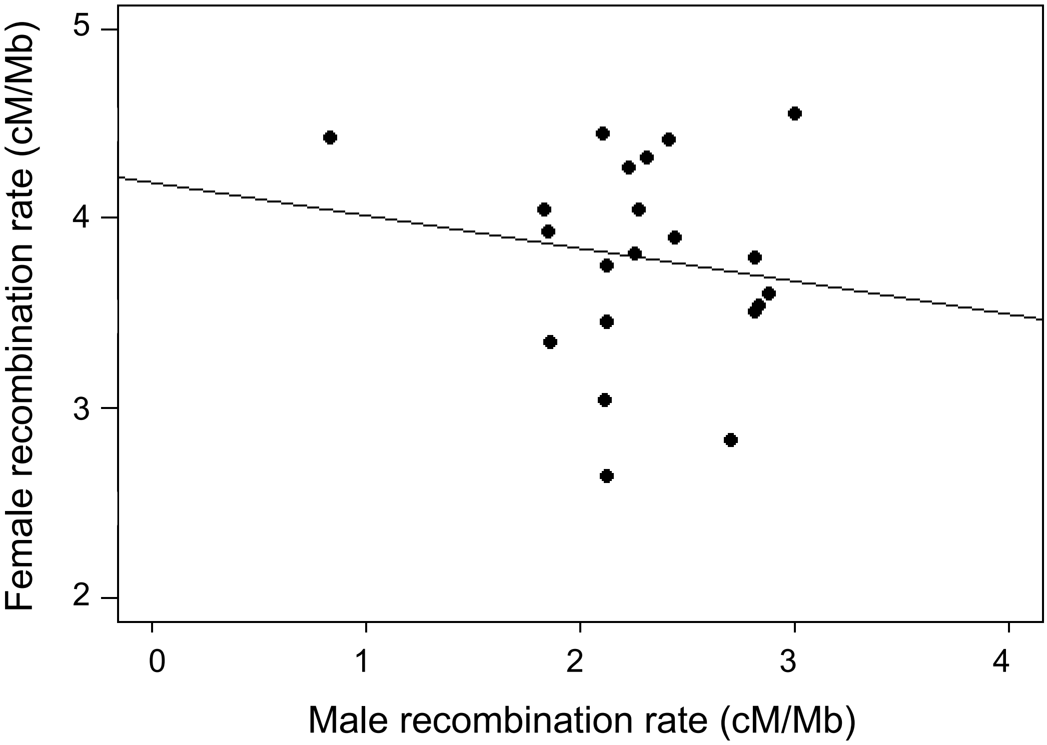

Supp. Fig. 2: Local recombination rates (cM / Mb) are not correlated between males and females for the 21 stickleback chromosomes. Line is the least-squares linear regression (r = 0.16).

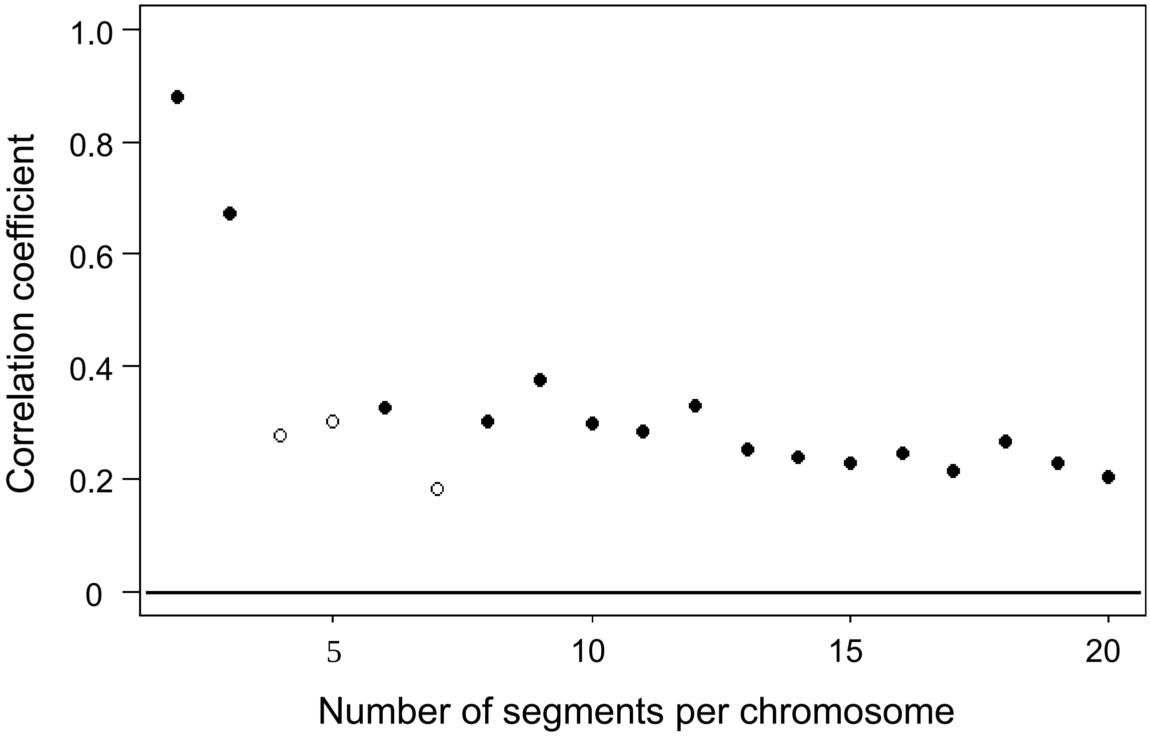

Supp. Fig. 3: Correlation between distribution of male and female COs along folded chromosomes at different scales. Chromosomes were divided into equal length segments and overall correlation calculated for fraction of male COs and fraction of female COs falling in segments. Black dots are scales for which positive correlations between male and female recombination are significant ($p < 0.05$)

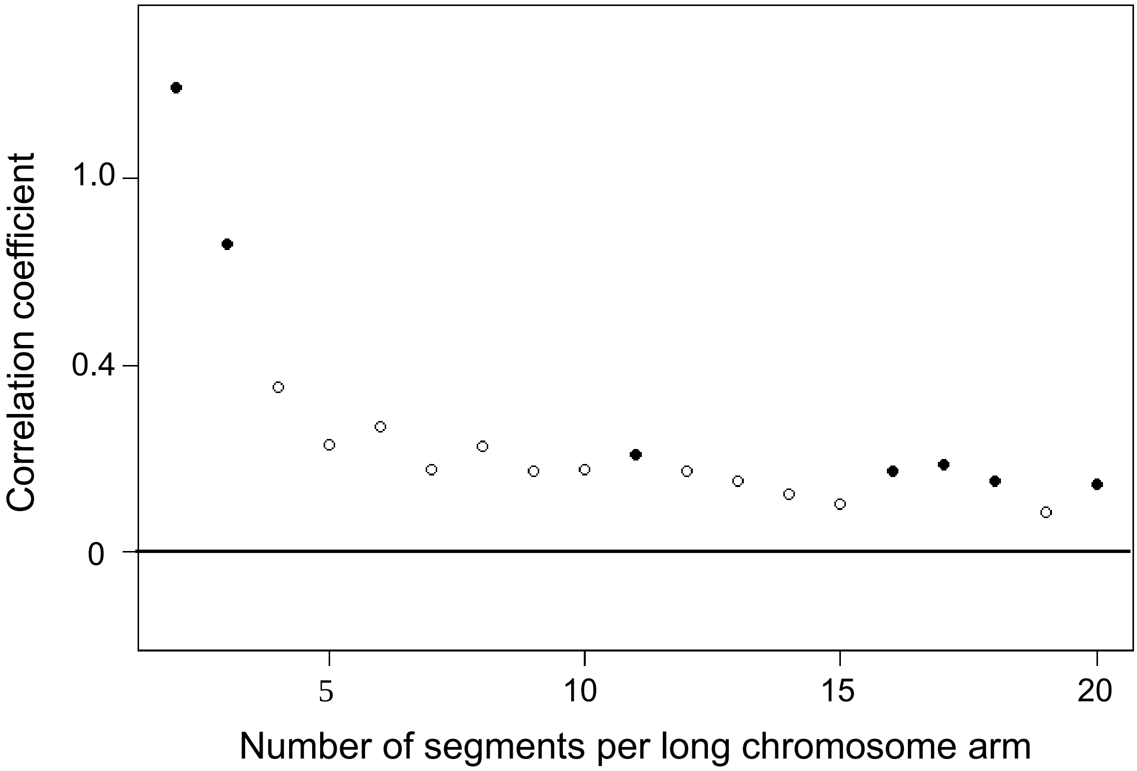

Supp. Fig. 4: Correlation between distribution of male and female COs along long chromosome arms at different scales. Arms were divided into equal length segments and overall correlation calculated for fraction of male COs and fraction of female COs falling in segments. Black dots are scales for which positive correlations between male and female recombination are significant ($p < 0.05$)

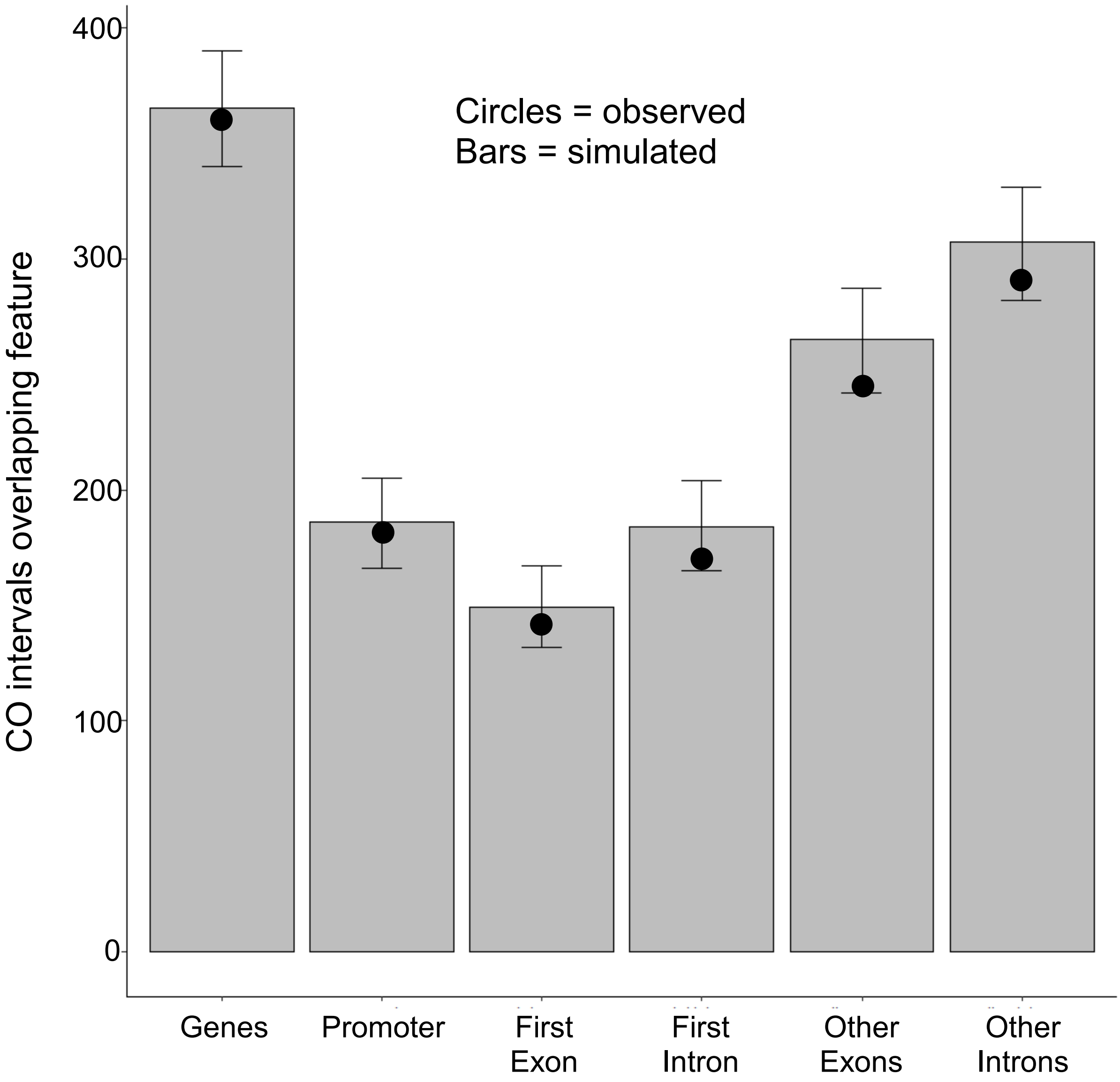

Supp. Fig. 5: Overlap between CO intervals and genetic features in sticklebacks. Black circles represent observed number of CO intervals overlapping feature. Bars represent results of 10,000 simulations in which intervals are randomly placed on chromosomes. Error bars for simulations are 95-percent confidence intervals. No observed result is significantly different from random expectations.

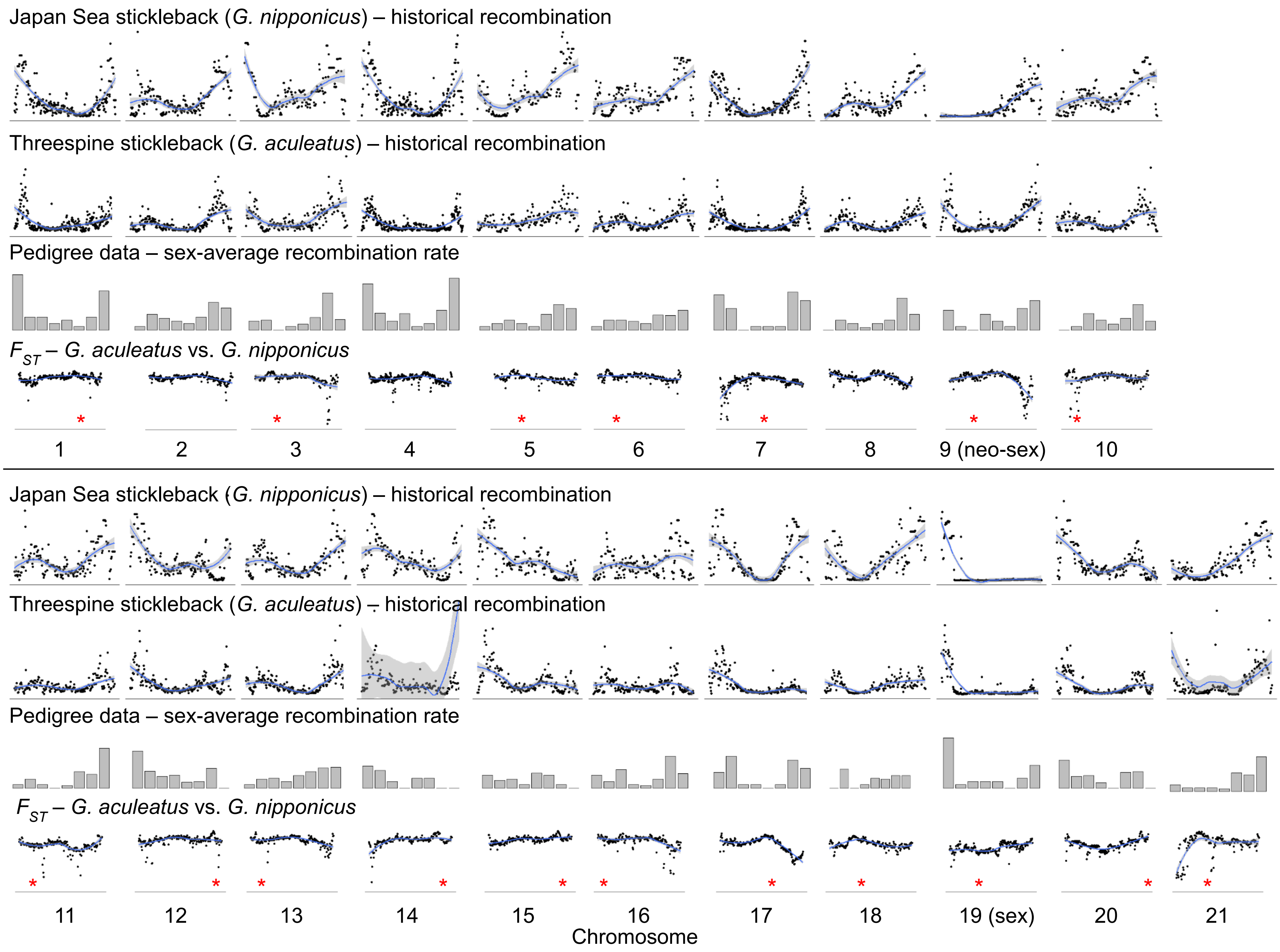

Japan Sea stickleback (G. nipponicus) – historical recombination
Threespine stickleback (G. aculeatus) – historical recombination
Pedigree data – sex-average recombination rate
F_ST – G. aculeatus vs. G. nipponicus
1
2
3
4
5
6
7
8
9 (neo-sex)
10
Japan Sea stickleback (G. nipponicus) – historical recombination
Threespine stickleback (G. aculeatus) – historical recombination
Pedigree data – sex-average recombination rate
F_ST – G. aculeatus vs. G. nipponicus
11
12
13
14
15
16
17
18
19 (sex)
20
21
Chromosome

Supp. Fig. 6: Comparison of historic population recombination rates for *G. nipponicus* (first row) and *G. aculeatus* (second row) with sex-average crossover data from hybrid crosses (third row) and genomic differentiation (F_{ST}) between species (fourth row). Historic recombination rate and F_{ST} data points represent averages over 100kbp non-overlapping sliding windows. Red asterisks represent centromere locations based on identification of centromere repeat motif in the *G. aculeatus* reference genome. Chromosome 19 is a sex chromosome in both species. Chromosome 9 is a neo-sex chromosome in *G. nipponicus*.

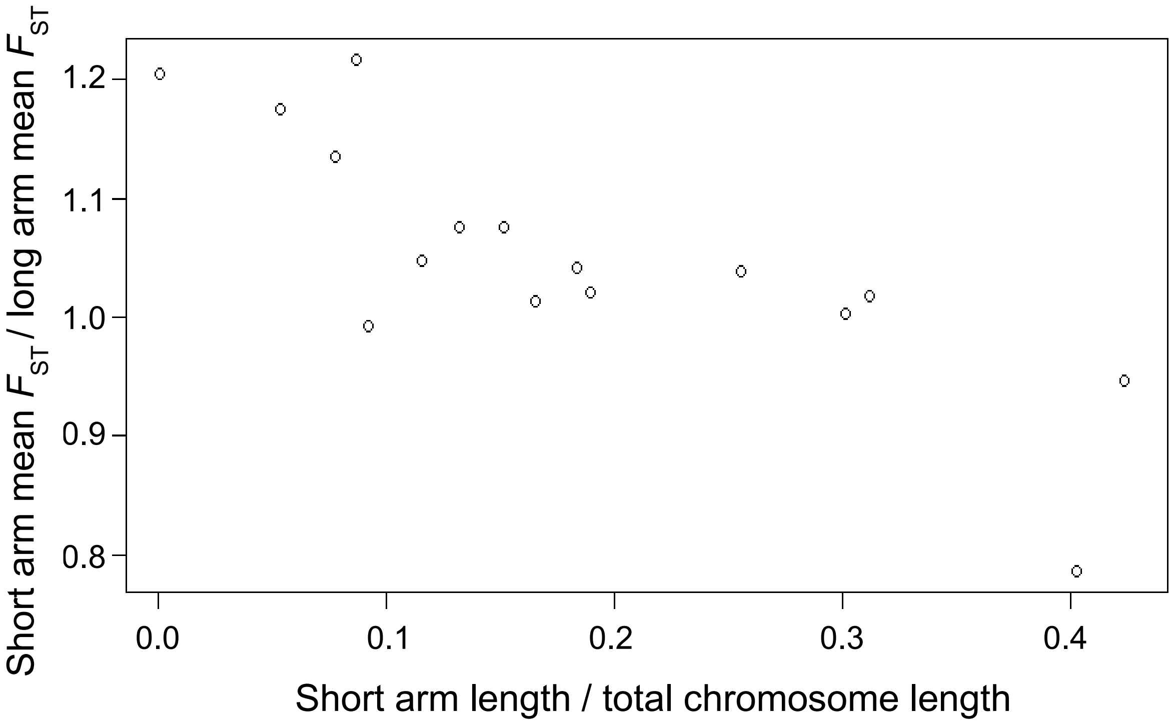

Supp. Fig. 7: Relative F_{ST} between *G. aculeatus* and *G. nipponicus* on short arms versus long arms for each chromosome plotted as a function of the fraction of the chromosome comprised of the short arm. Recombination is suppressed on the short arm in acrocentric chromosomes, resulting in increased relative genomic differentiation.
